# Compartment-specific pH monitoring in *Bacillus subtilis* using fluorescent sensor proteins: a tool to analyze the antibacterial effect of weak organic acids

**DOI:** 10.3389/fmicb.2013.00157

**Published:** 2013-06-18

**Authors:** Johan W. A. van Beilen, Stanley Brul

**Affiliations:** Department of Molecular Microbial Physiology, Swammerdam Institute for Life Sciences, University of AmsterdamAmsterdam, Netherlands

**Keywords:** bacterial spore formers, spores, spore germination, intracellular pH, GFP, pHluorin, weak organic acids, uncouplers

## Abstract

The internal pH (pH_i_) of a living cell is one of its most important physiological parameters. To monitor the pH inside *Bacillus subtilis* during various stages of its life cycle, we constructed an improved version (IpHluorin) of the ratiometric, pH-sensitive fluorescent protein pHluorin by extending it at the 5′ end with the first 24 bp of *comGA*. The new version, which showed an approximate 40% increase in fluorescence intensity, was expressed from developmental phase-specific, native promoters of *B. subtilis* that are specifically active during vegetative growth on glucose (P_ptsG_) or during sporulation (P_spoIIA_, P_spoIIID_, and P_sspE_). Our results show strong, compartment-specific expression of IpHluorin that allowed accurate pH_i_ measurements of live cultures during exponential growth, early and late sporulation, spore germination, and during subsequent spore outgrowth. Dormant spores were characterized by an pH_i_ of 6.0 ± 0.3. Upon full germination the pH_i_ rose dependent on the medium to 7.0–7.4. The presence of sorbic acid in the germination medium inhibited a rise in the intracellular pH of germinating spores and inhibited germination. Such effects were absent when acetic was added at identical concentrations.

## INTRODUCTION

The internal pH (pH_i_) of living cells plays a fundamental role in many chemical reactions. Many intracellular enzymes show optimal activity and stability in a narrow pH range near neutrality. Furthermore, in many organisms proton gradients are required for the greater part of ATP synthesis while uptake systems often depend on the proton gradient over the cell membrane (Krulwich et al., 1998, 2011; Slonczewski et al., 2009). In the model eukaryote *Saccharomyces cerevisiae*, pH_i_ was found to be a signal controlling growth (Orij et al., 2011). Gene expression as a response to glucose starvation was found to be mediated by changes in the pH_i_, through the protonation state-dependent binding of a transcription factor to membrane-associated phosphatidic acid (Young et al., 2010). In multicellular eukaryotes pH_i_ is thought to be important during growth and differentiation (Cruciat et al., 2010). In prokaryotic organisms, the relationships between pH_i_ and growth and development have not been studied extensively (Padan and Schuldiner, 1987).

Because of its various well-described differentiation modes, *B. subtilis* is generally considered to be the bacterial model organism for cellular differentiation. The best described mode of differentiation of this Gram-positive prokaryote is sporulation, with the pathways controlling sporulation understood in great molecular detail (Eichenberger et al., 2004; Steil et al., 2005; Wang et al., 2006). Germination is less well understood, but Keijser et al. (2007) have shown that this too, is a carefully orchestrated process. We reasoned that in analogy to eukaryotes, pH_i_ could be a global regulator, as well as an indicator of the metabolic and energetic state of the cell. To gain further insight in the putative pH_i_ dynamics of these differentiation processes, we studied the pH_i_ of the mother cell and fore-spore independently. During sporulation the development of the pH_i_ in the mother cell and the nascent fore-spore may also give insight in the level of independence of the two cells.

In *B. subtilis*, the pH_i_ of the developing pre-spore is generally assumed to drop to pH = 6.0–6.4 during sporulation (Magill et al., 1994). The drop in pH causes a decrease in activity of phosphoglycerate mutase (PGM), which catalyses the conversion of 3-phosphoglycerate (3-PGA) to 2-phosphoglycerate. The reduced activity of PGM causes the accumulation of 3-PGA in the pre-spore. *B. subtilis* spores are metabolically dormant and contain no measurable ATP or glucose that could act as energy source during spore germination (Singh et al., 1977; Magill et al., 1996). It is assumed that the accumulated 3-PGA serves as an initial carbon and energy source for the cell.

The cell’s pH_i_ can be measured with various methods. Ideally, intracellular pH measurements should be conducted in systems containing Good’s buffers (Good et al., 1966; Ferguson et al., 1980), to minimize the effect of the cell’s surrounding (unless desired). The probe used to measure pH should maintain accuracy over the pH range assessed. In addition, both the presence of the probe itself in a cell as well as the detection method applied should have minimal effect on cell physiology. Currently used techniques include the distribution of radiolabeled membrane-permeant weak acids, ^31^P nuclear magnetic resonance (NMR), fluorescent dyes (e.g., carboxyfluorescein, carboxyfluorescein diacetate, and succinimidyl ester; Ugurbil et al., 1978; Booth, 1985; Bulthuis et al., 1993; Magill et al., 1994; Breeuwer et al., 1996; Leuschner and Lillford, 2000). These methods have the advantage that no genetic modification is required and in the case of fluorescent dyes, single cell measurements are possible (Slonczewski et al., 2009). Weak acid dyes or reporters may alter the pH_i_ and are therefore difficult to use accurately, and may require many treatment and incubation steps before measurement. ^31^P NMR and radiolabeled compounds require extensive cell handling and high cell density, which also disturb cell physiology. Another useful method is the use of fluorescent proteins [green fluorescent protein (GFP) derivatives]. This does require the organism to be genetically accessible but allows direct, fast, and localized pH measurements. In our lab, we have successfully used ratiometric pHluorin (Miesenböck et al., 1998) for a number of years in *S. cerevisiae* (Orij et al., 2011; Ullah et al., 2012), and more recently also in *B. subtilis* (Ter Beek, 2009). However, the codon usage of pHluorin was not optimized for use in *B. subtilis*. Our initial experiments suggested that our results might benefit from an increase in fluorescence intensity. This might be achieved by improving translation initiation (Veening et al., 2004). We therefore fused the first eight amino acids of *comGA* to pHluorin (Veening et al., 2004), as this was shown to improve the signal strength of cyan fluorescent protein (CFP) and yellow fluorescent protein (YFP). The pH-dependent ratiometric fluorescent properties of IpHluorin were not affected by this fusion. To monitor the pH_i_ of both the mother cell and the pre-spore, ratiometric GFP-based IpHluorin was expressed from a number of developmental phase-specific native promoters of *B. subtilis* (Hilbert, 2004).

Expression of IpHluorin resulted in strong, compartmentalized, and cell type-specific signals. This allowed us to monitor the pH_i_ during growth and sporulation, in both pre-spore, mother cell and mature spore, as well as during spore germination. Effects of the addition of sorbic and acetic acid on the pH_i_ of germinating spores are described.

## MATERIALS AND METHODS

### STRAINS AND GROWTH CONDITIONS

For general purpose growth, *Escherichia coli* MC1061 and *B. subtilis* PB2 strains were grown in Lysogeny broth (LB). For fluorescence measurements, *B. subtilis* strains were grown in defined liquid medium (M3G; Keijser et al., 2007) buffered at pH = 5.5 or 6.4 with 80 mM 2-(*N*-morpholino)ethanesulfonic acid (MES), or at pH = 7.0 or 7.4 with 80 mM 3-(*N*-morpholino)propanesulfonic acid (MOPS). All cultures were grown at 37°C, under continuous agitation at 200 rpm. When required, the following antibiotics were added: kanamycin for strains carrying pDG148-derived plasmids; 10 μg/ml for *B. subtilis* strains, 50 μg/ml for *E. coli* strains, spectinomycin for strains carrying pSG1729-derived plasmids or genomic inserts (50 μg/ml). The strains used in this study are listed in **Table 1**.

**Table 1:** Strains used in this study.

Strains	Genotype	Reference or construction
*E. coli* MC1061	Cloning host; F^-^ *araD139 (ara-leu)7696 (lac)X74 galU galK hsdR2 mcrA mcrB1 rspL*	Casadaban and Cohen (1980)
*Bacillus subtilis*		
PB2	*trp*2C; 168 wild-type	C.W. Price
PB2 pDG148	*trp*2C; pDG148**	This work
PB2 Pxyl-pHluorin	*trp*2C; *amyE3*′* spc Pxyl–pHluorin amyE5*′	This work
PB2 Pxyl-IpHluorin	*trp*2C; *amyE3*′ *spc Pxyl–IpHluorin amyE5*′	This work
PB2 pDG-pHluorin	*trp*2C; pDG-pHluorin	This work
PB2 pDG-IpHluorin	*trp*2C; pDG-IpHluorin	This work
PB2 PptsG-IpHluorin	*trp*2C; *amyE3*′ *spc PptsG–IpHluorin amyE5*′	This work
PB2 PspoIIA-IpHluorin	*trp*2C; *amyE3*′ *spc PspoIIA–IpHluorin amyE5*′	This work
PB2 PspoIIID-IpHluorin	*trp*2C; *amyE3*′ *spc PspoIIID–IpHluorin amyE5*′	This work
PB2 PsspE-IpHluorin	*trp*2C; *amyE3*′ *spc PsspE–IpHluorin amyE5*′	This work

### SPORULATION OF *B. subtilis* STRAINS

Spores of *B. subtilis* were prepared by glucose depletion of defined liquid medium (M3S, which is M3G without sodium glutamate), at pH = 7.0. Cultures were incubated for 4 days at 37°C under continuous agitation (200 rpm). Spores were harvested and purified by extensive washing with MilliQ water at 4°C. The spore crops were inspected by phase-contrast microscopy and were free (>99%) of vegetative cells, germinating spores, and debris. Spores were stored for up to 1 week in MilliQ water at 4°C at optical density (OD)_600_ = 1.

### CLONING OF PROMOTER FUSIONS WITH *IpHluorin*

Our initial experiments suggested that the accuracy of pH measurements might benefit from increased expression of pHluorin. To improve translation efficiency, the first 24 bp of *comGA*, with an ATG start codon, were fused to *pHluorin* by a polymerase chain reaction (PCR) with Pfu polymerase using primers IpHlu_2010_FW and IpHlu_2010_RV. This sequence was subsequently extended with a standard Shine–Dalgarno (SD) region (AAGGAGGAAGCAGGT; Joseph et al., 2001) using primers IpHlu_pDGA_FW. This SD-improved *pHluorin* (*IpHluorin*) was inserted between the *Hin*dIII and *Sal*I sites of pDG148. This construct, pDG-IpHluorin, was transformed into *B. subtilis* PB2 and compared with PB2 carrying pDG-pHluorin to analyze expression levels and pH-dependent characteristics of pHluorin and IpHluorin. Also, a xylose-inducible, genome-integrated expression system was constructed. To this end, *IpHluorin* was inserted in pSG1729, between the *Avr*II and *Hin*dIII sites, thereby replacing GFP and placing *IpHluorin* under control of the xylose-inducible P_xyl_ promoter.

To monitor the pH_i_ of *B. subtilis* for extended periods of time in different phases of its life cycle, without the need for externally supplied expression inducers, the promoter region of several growth phase-specific genes (P_ptsG_, for vegetative cells growing on glucose, P_spoIIA_, specific for pre-septum, sporulating cells, P_sspE_, a fore-spore-specific gene, and P_spoIIID_, a mother cell-specific promoter) of *B. subtilis* were selected for their expression levels (Steil et al., 2005; Veening et al., 2006a). Approximately 500 bp upstream of the start codon were selected for cloning. By standardizing the SD region, we aimed to increase and standardize the expression levels of poorer promoter sites (Ozbudak et al., 2002; Botella et al., 2010). The promoter and *SD-IpHluorin* sequences were fused by a PCR and inserted in pSG1729, between the *Avr*II and *Hin*dIII sites, thereby replacing the GFP and placing IpHluorin under control of a *B. subtilis* promoter. All enzymes used were obtained from Fermentas (Thermo Fisher Scientific).

*Bacillus subtilis* PB2 was used as target for our transformations. *B. subtilis* cells were made transformation-competent as described before (Kunst and Rapoport, 1995). The newly constructed plasmids were integrated in the *amyE* locus as described (Lewis and Marston, 1999). All plasmids and oligonucleotides used in this study are listed in **Tables 2 and 3.**

**Table 2:** Plasmids used in this study.

Plasmid	Genotype	Reference or construction
pDG148	*bla ble kan lacI Pspac*	Stragier et al. (1988)
pDG-pHluorin	(pDG148); *pHluorin*	
pDG-IpHluorin	(pDG148); *IpHluorin*	This work
pSG1729	*bla amyE3′ spc Pxyl–gfpmut1′*amyE5*′*	Lewis and Marston (1999)
pSG-pHluorin	*bla amyE3*′ *spc Pxyl–pHluorin amyE5*′	This work
pSG-IpHluorin	*bla amyE3*′* spc Pxyl–IpHluorin amyE5*′	This work
pSGP_ptsG_-IpHluorin	*bla amyE3*′* spc PptsG–IpHluorin amyE5*′	This work
pSGP_spoIIA_-IpHluorin	*bla amyE3*′* spc PspoIIA–IpHluorin amyE5*′	This work
pSGP_spoIIID_-IpHluorin	*bla amyE3*′* spc PspoIIID–IpHluorin amyE5*′	This work
pSGP_sspE_-IpHluorin	*bla amyE3*′* spc PsspE–IpHluorin amyE5*′	This work

**Table 3:** Oligonucleotides used in this study.

Oligonucleotide	Sequence (5′–3′)	Remarks
IpHlu2010_FW	ATGGATTCAATAGAAAAGGTAAGC**ATG**AGTAAAGGAGAAGAAC	Forward primer for *IpHluorin*
IpHlu148_RV	CGACunderbarGTCGACTTTATTTGTATAGTTCATCCATGCC	Reverse *IpHluorin* primer for pDG148
IpHlu148A_FW	CCCunderbarAAGCTTAA*GGAGGA*AGCAGGT**ATG**GATTCAATAGAAAAG	Forward *IpHluorin* primer for pDG148
IpHlu1729A_FW	ACGCunderbarCCTAGG**ATG**GATTCAATAGAAAAGGTAAGC	Forward *IpHluorin* primer for pSG1729
IpHlu2010_RV	CCCunderbarAAGCTTTTATTTGTATAGTTCATCCATGCCATG	Reverse *IpHluorin* primer for pSG1729
PptsG_FW	ACGCunderbarCCTAGGGAAAGTAAATAAGGAAAGTGTCAAC	5′ end of *P*_ptsG_
PptsG_IpHlu_RV	CATACCTGCTT*CCTCCT*TTTTTACTAGTCTGACCTTAC	3′ end of *P*_ptsG_
PptsG_IpHlu_FW	GTAAGGTCAGACTAGTAAAAA*AGGAGGA*AGCAGGT**ATG**	3′ of *P*_ptsG_ and 5′ of *IpHluorin*
PspoIIAA_FW	ACGCCunderbarCTAGGCCATAGCGGTTGTATTC	5′ end of *P*_spoIIA_
PspoIIAA_IpHlu_RV	CATACCTGCTT*CCTCCT*TGATATGATCGGATAATGAGTGTTTC	3′ end of *P*_spoIIA_
PspoIIAA_IpHlu_FW	GAAACACTCATTATCCGATCATATCA*AGGAGG*AAGCAGGT**ATG**	3′ of *P*_ptsG_ and 5′ of *IpHluorin*
PspoIIID_FW	ACGCunderbarCCTAGGCTGACCATTGAGATGAATAAAG	5′ end of *P*_spoIIID_
PspoIIID_IpHlu_RV	**CAT**ACCTGCTT*CCTCCT*TAAAATGGATGTGAGAAGTGTGAAATGAG	3′ end of *P*_spoIIID_ and 5′ of IpHluorin
PspoIIID_IpHlu_FW	CTCATTTCACACTTCTCACATCCATTTTA*AGGAGG*AAGCAGGT**ATG**	3′ of *P*_ptsG_ and 5′ of *IpHluorin*
PsspE_FW	ACGCunderbarCCTAGGTGAACATTAATGCGAAAGCATTG	5′ end of *P*_sspE_
PsspE_IpHlu_RV	**CAT**ACCTGCTT*CCTCCT*TCGGTCATTAGAATGTCCAG	3′ end of *P*_sspE_
PsspE_IpHlu_FW	CTGGACATTCTAATGACCGA*AGGAGG*AAGCAGGT**ATG**	3′ of *P*_sspE_ and 5′ of *IpHluorin*

*Underlined bases: restriction enzyme recognition sites; bases in italics: Shine–Dalgarno sequence; bold bases: start codon.*

### CALIBRATION OF *IpHluorin*

*Bacillus subtilis* PB2 containing either pDG148, pDG-pHluorin or pDG-IpHluorin were grown to exponential phase in M3G at pH 7.0 containing 10 μg/ml kanamycin. Bacterial growth and expression levels of ratiometric pHluorin and IpHluorin were monitored in a FluoStar Optima (BMG Labtech, Germany) for 3 h after addition of 0–1 mM isopropyl β-D-1-thiogalactopyranoside (IPTG).

For calibration of the pH, expression of ratiometric pHluorin and IpHluorin was induced for 2.5 h by the addition of 1 mM IPTG. At OD_600_ = 0.4 the cells were centrifuged and resuspended in buffers with pH values ranging from 5.0 to 8.5 prepared from 0.1 M citric acid and 0.2 M K_2_HPO_4_. The intracellular and extracellular pH were equilibrated by the addition of 1 μM valinomycin and 1 μM nigericin (Breeuwer et al., 1996). Cells were transferred to black-walled microtiter plates and incubated at 37°C in a FluoStar Optima. OD_600_ was measured before start of the experiment. The ratio of emission intensity at 510 nm resulting from excitation at 390 and 470 nm (with photomultiplier gain set to 2,000) was calculated as described previously (Orij et al., 2011). Fluorescence and OD_600_ were monitored for 30 min, with measurements taken every 5 min. Calibration curves for pHluorin and IpHluorin were identical, with only minor fluctuations in fluorescence in time observed with pHluorin at pH = 8.5. From this, we concluded that the intracellular and extracellular pH had equilibrated rapidly. *B. subtilis* PB2 carrying pDG148 was measured for background fluorescence. Background fluorescence was subtracted at individual wavelengths before calculating the ratio. The calibration curve was determined by fitting the data of three independent biological replicates, each consisting of three technical replicates, with a polynomial curve of the third order.

### BATCH MEASUREMENTS OF pH_i_ DURING SPORULATION, GERMINATION, AND OUTGROWTH

To monitor pH_i_ during growth and sporulation, all *B. subtilis* strains, wild-type (WT) (PB2) and those with IpHluorin fused to endogenous promoters were grown as described, in M3S without antibiotics, pH 7.0, to an OD_600_ = 0.1 in an incubator at 37°C under continuous agitation (200 rpm). Cell suspensions were diluted twofold by adding 50 μl of culture to 50 μl of medium in black microtiter plates which were then monitored in a FluoroStar Optima BMG (Labtech, Germany) at 37°C. OD_600_ and pH measurements were taken every 10 min for 48 h. The plates were shaken (200 rpm) in between measurements thus ensuring optimal growth (Ter Beek, 2009). For spore germination, washed spores were heat activated (30 min, 70°C, then cooled on ice) and subsequently mixed 1:1 with 2× concentrated M3 with or without glucose, containing weak organic acid (WOA) in predetermined concentrations. To trigger germination, 5 μl 20× concentrated AGFK (10 mM L-asparagine, 10 mM D-glucose, 1 mM D-fructose, 1 mM KCl; Wax and Freese, 1968) was added. Microtiter plates were placed in a FluoStar Optima (BMG Labtech, Germany) at 37°C and shaken between measurements (200 rpm). Growth was monitored for 2–12 h, with pH and OD_600_ measurements taken every 10 min.

### MICROSCOPY

To verify if expression of IpHluorin was correctly localized, *B. subtilis* cells were cultured as described above for batch measurements at pH = 7.0. All strains were grown as described to exponential phase or for 16–24 h to observe sporulating cells. Cells were immobilized on 1% agarose (Koppelman et al., 2004), and photographed with a CoolSnap *fx* (Photometrics) charge-coupled device (CCD) camera mounted on an Olympus BX-60 fluorescence microscope through an UPLANFl 100×/1.3 oil objective (Japan) with a 41017 - Endow GFP/EGFP Bandpass filter (Chroma Technology Corp., Bellows Falls, VT, USA).

## RESULTS

### IMPROVED EXPRESSION OF pHluorin

**FIGURE 1:**
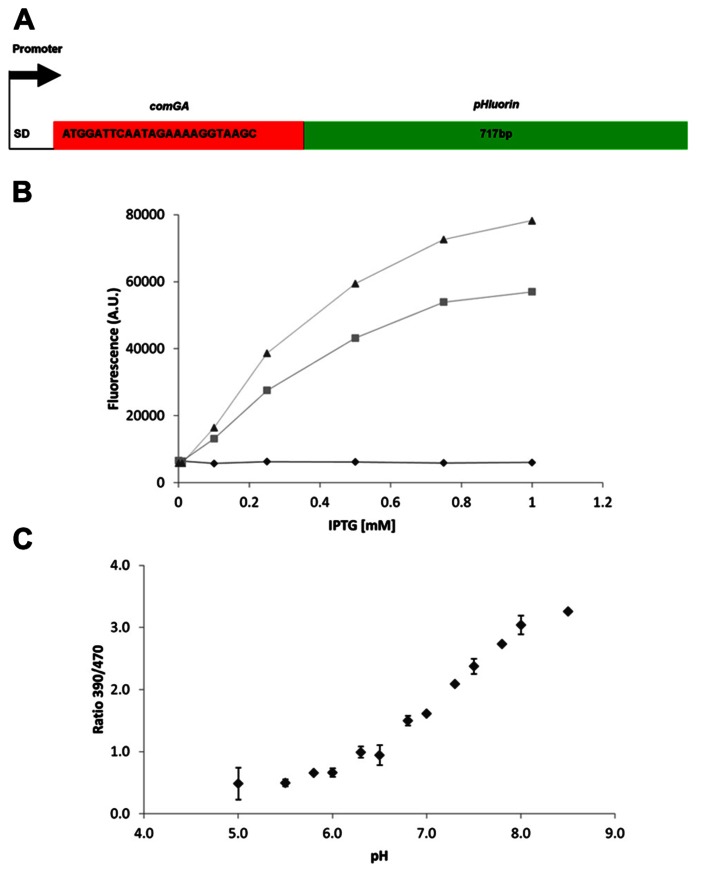
**Improved expression of pHluorin in *B. subtilis*.** **(A)** Schematic overview of the improved pHluorin construct. SD – Shine–Dalgarno sequence. comGA – the first 24 bp of *comGA*, with the start codon converted to ATG. **(B)** Expression levels of pHluorin and improved pHluorin after induction with IPTG. Fluorescence (AU, arbitrary units) at 510 nm from excitation at 390 nm was measured after 2.5 h of induction and normalized to OD_600_. Diamonds, control (pDG148); squares, pHluorin (pDG-pHluorin); triangles, IpHluorin (pDG-IpHluorin). **(C)** Calibration curve for IpHluorin. Error bars indicate standard deviations (*n* = 3).

Many microorganisms have an internal (cytosolic and/or mitochondrial) pH between 7 and 8 (Orij et al., 2009; Slonczewski et al., 2009) during optimal growth and maintaining pH homeostasis is of vital importance for most, including *B. subtilis* where pH_i_ differences have been inferred for its various developmental phases. We now used the pH-sensitive GFP pHluorin, developed for yeast (Miesenböck et al., 1998), to directly measure on-line the pH_i_ dynamics in *B. subtilis*. Codon usage of this GFP was not optimized for *B. subtilis* and our initial experiments suggested that expression might be improved. It was shown previously that addition of the first eight amino acids of *comGA* improved translation initiation efficiency of CFP and YFP in *B. subtilis* (Veening et al., 2004). We used this approach to construct improved pHluorin (IpHluorin; **Figure 1A**).

To analyze fluorescence intensity of *Bacillus* cells harboring pDG148, pDG-pHluorin, or pDG-IpHluorin cells were transferred to microtiter plates containing 0–1 mM IPTG to induce expression. Cell growth was monitored for 3 h, together with fluorescence emission at 510 nm upon excitation at 390 and 470 nm. The three strains compared had identical growth rates (not shown). Fluorescence intensity after 2.5 h is shown in **Figure 1B**, and depended on the concentration of IPTG. Cells expressing IpHluorin had the highest signal intensity at 1 mM IPTG, approximately 40% higher than pHluorin under the same conditions. The most important characteristic of pHluorin is its shift in excitation wavelength depending on the pH of its surroundings. To establish if the addition of eight amino acids at the N-terminus would alter these characteristics, calibration curves for pHluorin and IpHluorin were made (**Figure 1C** for IpHluorin, not shown for pHluorin). Both growth rate and the ratiometric characteristics were similar and allowed reliable pH_i_ readings in a range between pH 5 and 8.5 in live *B. subtilis* cultures.

### THE INTERNAL pH VARIES WITH GROWTH PHASE

**FIGURE 2:**
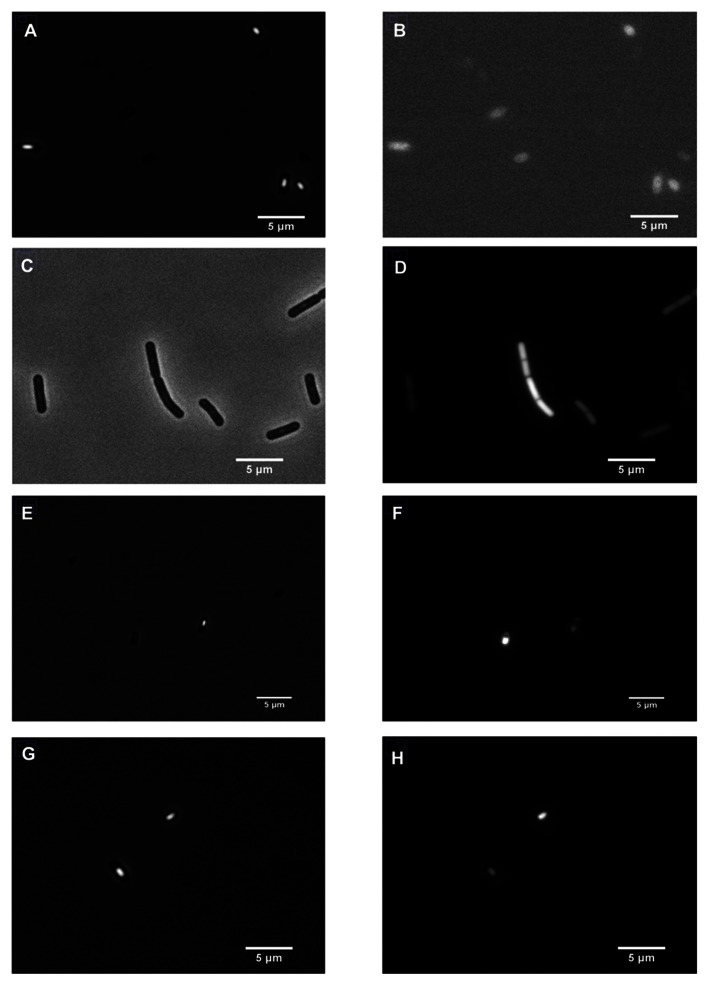
**Expression of IpHluorin in *B. subtilis* PB2.**> *Left panels*: phase-contrast images; *right panels*: corresponding fluorescent signals. **(A,B)** Non-transformed cells; **(C,D)** PptsG-IpHluorin-expressing cells; **(E,F)** sporulating cells expressing PspoIIID-IpHluorin; **(G,H)** sporulating cells expressing PB2 PsspE-IpHluorin.

The cytosolic pH is a crucial parameter for bacteria because it modulates the activity of many enzymes (Vojinovic and Von Stockar, 2009) and in many species plays a crucial role in generating the proton-motive force (Shioi et al., 1980; Slonczewski et al., 2009). To monitor pH_i_ during various stages of growth in *Bacillus*, we fused promoters of strongly expressed, growth phase-specific genes to IpHluorin. This allowed us to measure pH_i_ of *B. subtilis* without addition of inducers such as IPTG or xylose (**Figures 2A–H**). The selected promoters and their specific expression phase are shown in **Table 4.**

**Table 4:** Promoters used for IpHluorin expression.

Promoter	Regulator	Corresponding growth phase
P_ptsG_	σ^A^	Growth on glucose (Botella et al., 2010)
P_spoIIA_	Spo0A, σ^F,G,H^	Early sporulation (Wang et al., 2006)
P_spoIIID_	σ^E^	Early sporulation, mother cell-specific (Wang et al., 2006)
P_sspE_	σ^G^	Late sporulation, spore-specific (Wang et al., 2006)

**FIGURE 3:**
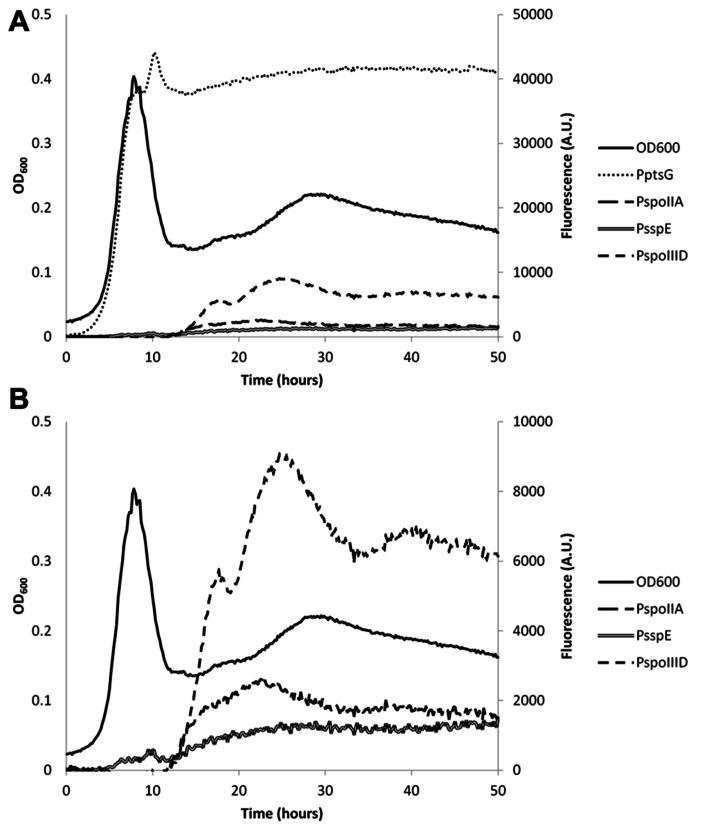
**Representative example of the expression levels of IpHluorin from different promoters during different growth stages.** **(A)** Fluorescence levels from excitation at 390 nm and emission at 510 nm from different promoters used. **(B)** Identical to **(A)**, but without P_ptsG_-IpHluorin. Expression of IpHluorin from sporulation-specific promoters starts only after the drop in OD_600_.

**FIGURE 4:**
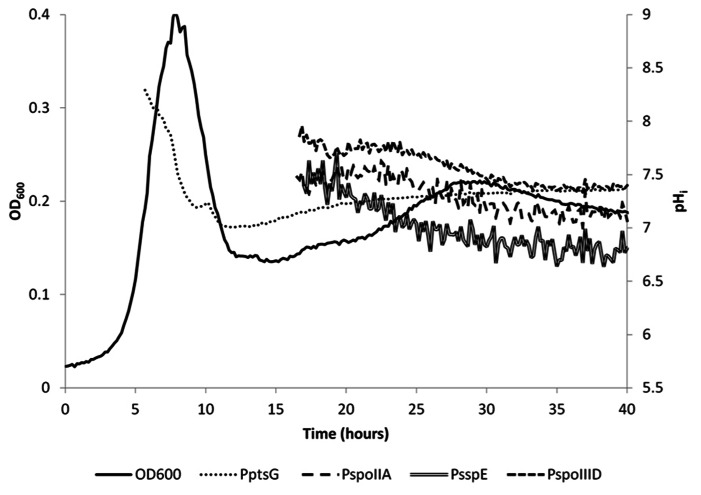
**Internal pH as measured with IpHluorin expressed from different promoters.** The pH is calculated from when the 390 nm channel exceeded 1,000 units. pH from the spore-specific promoters is calculated from approximately 17 h onward. Lines represent actual data from a representative example.

To monitor the pH_i_ during growth in minimal medium with glucose as the only carbon source, we used the promoter of *ptsG*, which encodes the glucose-specific enzyme II of the carbohydrate:phosphotransferase system to drive *IpHluorin* expression. P_ptsG_ is a strong promoter during vegetative growth on glucose (Botella et al., 2010). Expression of IpHluorin from the P_ptsG_ promoter follows the growth curve closely (**Figure 3A**). When the cells die or move into stationary phase (after 7.5 h), the signal intensity remains high and stable. The sporulation-specific promoters (**Figure 4B**) are activated after the drop in OD_600_, signifying the onset of sporulation.

The pH_i_ of *B. subtilis* reaches its highest value of around 8 during exponential growth. This value is in agreement with earlier reported values ranging from pH = 7.8 to 8.1 (Setlow and Setlow, 1980; Magill et al., 1994). At the drop in OD_600_, cells either die or differentiate and initiate sporulation or remain in stationary phase. This was accompanied by an apparent steep decrease in pH_i_, to 7.0 in vegetative cells expressing IpHluorin from P_ptsG_. Likely, this at least partially is indicative for cell lysis as a strong fluorescent signal could also be detected in the medium after spinning down the cells. Additionally, it is possible that morphological changes of the cell affect their optical properties. Sporulating cells are, for instance, smaller than exponentially growing cells. Hence, after sporulation commences, the pH values observed with P_ptsG_-IpHluorin can no longer be considered an accurate estimate of the intracellular pH in vegetative cells. Apart from aberrant values due to cell lysis, the P_ptsG_-driven IpHluorin may also get trapped in sporulating cells so that the observed pH from P_ptsG_-driven IpHluorin is the average of sporulating and non-sporulating cells as well as the medium. Subsequently, the OD_600_ rose again slowly and the apparent pH increased to 7.4 (**Figure 3**). We do not know from which cells this signal originates as it may represent the average of various differentiation types, all expressing IpHluorin. To deconvolute these signals, single cell measurements are needed.

### SPORULATION-SPECIFIC EXPRESSION OF IpHluorin

Sporulation of *B. subtilis* is a well-described, carefully orchestrated process where a number of different sigma factors are activated during subsequent stages (Wang et al., 2006). It has been reported that the pH_i_ of *Bacillus* spores is lower than that of vegetative cells (Magill et al., 1994). We set out to measure the pH in spores and at what stage in sporulation the drop in pH starts and when the pH would rise again during germination. For this purpose, we constructed strains with early and late (pre)spore-specific expression of IpHluorin. We selected promoters that would be active in the pre-spore and mother cell at different times during sporulation to monitor pH_i_ of both cells separately. Expression from P_spoIIA_, P_spoIIID_, and P_sspE_ starts after the drop in OD_600_. The surviving cells may prepare for diauxic growth or sporulation (Veening et al., 2008). This characteristic allowed us to measure differences in pH_i_ in both mother cell and pre-spore in the subpopulation that initiates sporulation. Expression levels from P_spoIIA_, P_spoIIID_, and P_sspE_ are lower than of P_ptsG_, but are still reliable and strong enough to allow pH monitoring (**Figures 3A,B**). For the sporulation-specific promoters, a cut-off of 1,000 arbitrary units in the 390 to 510 nm fluorescence channel was used for pH_i_ calculations.

SpoIIA is activated by high levels of activated Spo0A and its presence was shown to be a reliable indicator for cells that initiate sporulation (Veening et al., 2005). Indeed, a fluorescent signal of a GFP reporter under control of the SpoIIA promoter can be found in both mother cell and fore-spore (our unpublished observations; Veening et al., 2006b). Expression of genes in the mother cell regulated by P_spoIIID_ follows that of those regulated by P_spoIIA_, as expected, but because expression levels of P_spoIIID_-controlled IpHluorin are higher, reliable pH_i_ measurements can be obtained earlier with the latter. Initially, the sporulating subpopulation had a pH_i_ that closely resembled the pH_i_ of exponentially growing cells measured with the P_ptsG_-IpHluorin strain. The mother cell (P_spoIIID_-IpHluorin) had a pH of 7.8 after 17 h of incubation. IpHluorin expressed from the spore-specific promoter P_sspE_ revealed pH values of 7.4. The mother cell-specific expression of IpHluorin from the *spoIIID* promoter decreased after 25 h of culture. At that time point and from then onward, an apparent decrease measured with the mother cell-specific promoter driving IpHluorin expression was observed. This data, however, may at least partially be influenced by mother cell lysis and release of IpHluorin into the medium. The inferred pH at 40 h of culture closely resembled medium pH, corroborating this notion.

The decrease in pH_i_ in the fore-spore (P_sspE_-IpHluorin) drops below the medium pH and its fluorescent signal can clearly be observed inside maturing spores (**Figure 2H**). Noticeably, spores have a very low water activity and optical properties dissimilar from vegetative cells, which may obscure the pH as defined as the number of free protons (Sunde et al., 2009).

**FIGURE 5:**
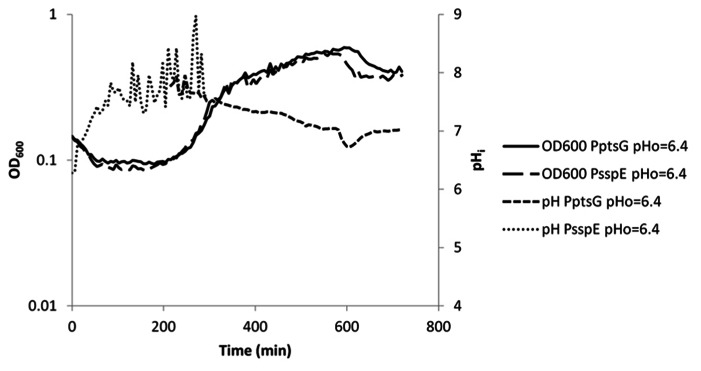
**Internal pH during spore germination and outgrowth.** IpHluorin accumulated in the spores (from expression controlled by P_sspE_) allows pH measurements from *t* = 0 to approximately 300 min. Expression of IpHluorin from P_ptsG_ allows calculation of the pHi from approximately 200 min. Data are from a representative example of germination and outgrowth of *B. subtilis* spores at an external pH of 6.4.

Our data indicates that at 17.5 h of culture, the pH_i_ of the fore-spore is 7.4, as reported by P_sspE_-IpHluorin. After 40 h, the pH value of 6.8 reported by P_sspE_-IpHluorin is approaching the reported value for *Bacillus* spores (pH_i_ = 6.0 ± 0.3; Barton et al., 1980; Setlow and Setlow, 1980; Magill et al., 1994, 1996). Likely, because at this time point the population is still a mix of some fore-spore-containing cells as well as many free spores, the observed pH is slightly higher than the reported values for isolated *Bacillus* spores. Corroborating this, when we washed and isolated the spores our pHluorin-based measurement of the pH_i_ of *B. subtilis* spores also indicated values around 6.0 ± 0.3 (see, e.g., pHi data of time point 0 obtained with IpHluorin driven by P_sspE_ in **Figure 5** and beyond).

### INTERNAL pH DURING SPORE GERMINATION AND OUTGROWTH

As described above, IpHluorin expressed from P_sspE_ accumulates in mature spores. Germination and outgrowth were monitored used *B. subtilis* PB2 P_sspE_-IpHluorin and *B. subtilis* PB2 P_ptsG_-IpHluorin. When germination is triggered by addition of a mixture of asparagine, glucose, fructose and potassium (AGFK), the OD_600_ of the spore crop drops, because the refractile spores turn phase-dark, due to water uptake. Simultaneously, the spore’s pH_i_ rises. Depending on the medium pH, the pH_i_ rises to 7.0–7.4 (**Figure 5** and our unpublished observations for germination at pH 7.4, respectively). In the case of germination at pH = 6.4, this indicates the establishment of a pH gradient. Not all spores germinate at the same time, and significant heterogeneity can be observed in the timing of germination and outgrowth (Smelt et al., 2008). Since this is a mixed population, consisting of phase-bright and germinating spores, the actual pH change in individual germinating spores may differ.

During the lag phase between germination and outgrowth, the *ptsG* promoter is activated. Parallel expression of IpHluorin from this promoter shows that the pH measured this way lies between 7.5 and 7.8. This range of pH values is maintained during exponential growth. A generally observed slow decrease in pH may be due to acidification of the medium by acetic acid or CO_2_ (Russell and Diez-Gonzalez, 1998; Orij, 2010). After approximately 600 min, there is a sudden drop in pH and OD_600_ as described above (**Figure 5**).

Germination with medium pH = 7.4 shows a more rapid decrease in OD_600_ and an equally faster rise in pH_i_. Also, when outgrowth commences, pH_i_ of these cells is higher, but follows a similar trend as with medium at pH = 6.4.

### INTERNAL pH DURING SPORE GERMINATION WITH WEAK ACID STRESS

Dormant spores are highly resistant to antimicrobial treatment, but also metabolically inert (Brul and Coote, 1999). When germination is triggered, the spore becomes more sensitive. Also, it has been observed that germination of *Bacillus* spores can be inhibited by various preservatives (Cortezzo et al., 2004; Van Melis et al., 2011). When spores start to germinate, they release protons and the pH_i_ rises. Also, during this stage water is taken up and metabolism should be restarted. These processes might be a target moment for WOAs to halt outgrowth of the germinating spore.

**FIGURE 6:**
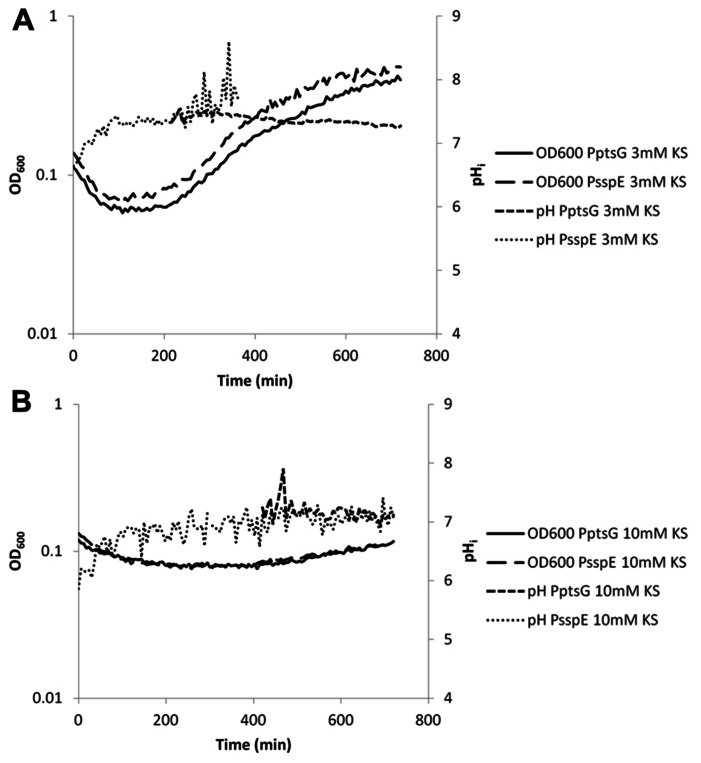
**Internal pH during spore germination and outgrowth.** IpHluorin accumulated in the spores (from expression controlled by P_sspE_) allows pH measurements from *t* = 0 to approximately 300 min. Expression of IpHluorin from P_ptsG_ allows calculation of the pHi from approximately 200 min. Data are from a representative example. **(A)** Ge rmination and outgrowth of *B. subtilis* spores at an external pH = 6.4 with 3 mM KS. **(B)** Germination and outgrowth of *B. subtilis* spores at an external pH = 6.4 with 10 mM KS.

**FIGURE 7:**
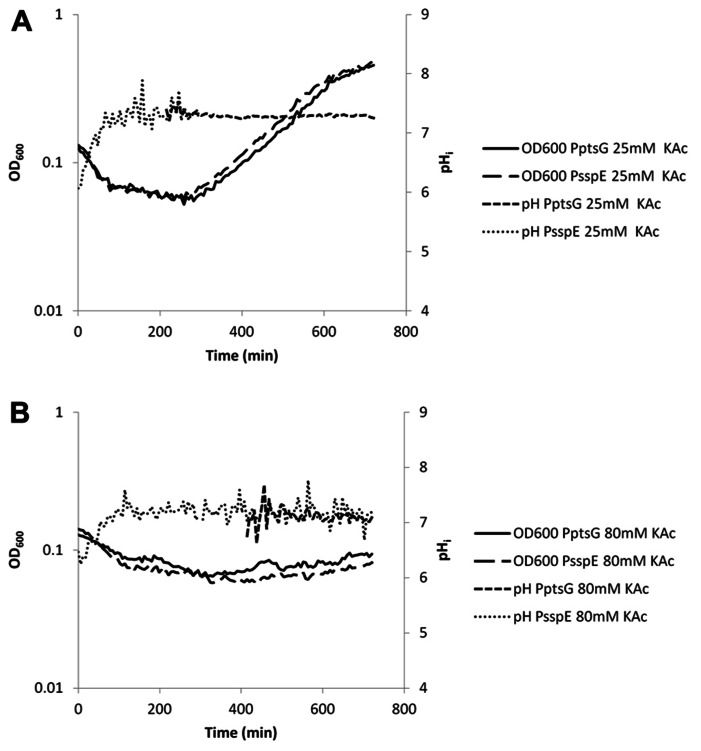
**Internal pH during spore germination and outgrowth.** IpHluorin accumulated in the spores (from expression controlled by P_sspE_) allows pH measurements from *t* = 0 to approximately 300 min. Expression of IpHluorin from P_ptsG_ allows calculation of the pHi from approximately 200 min. Data are from a representative example. **(A)** Germination and outgrowth of *B. subtilis* spores at an external pH = 6.4 with 25 mM KAc. **(B)** Germination and outgrowth of *B. subtilis* spores at an external pH = 6.4 with 80 mM KAc.

Acetic and sorbic acid are amongst the most commonly used food preservatives (Stratford et al., 2009; Ter Beek and Brul, 2010; Ullah et al., 2012). While both WOAs have a similar p*K*_a_ value, sorbic acid is clearly the more potent antimicrobial compound. We compared the effects of sorbic and acetic acid on germination and outgrowth by using concentrations of both acids that had a similar effect on growth rate (Ter Beek, 2009). Low concentrations of both acids reduced the exponential growth rate by approximately 50%. Spores germinating in medium (pH = 6.4) with 3 mM K-sorbate had a decreased rate of pH_i_ increase. In controls the pH_i_ increase between the start of germination and *t* = 90 min was 1.4 units whilst with 3 mM K-sorbate this was 0.7 units. At the onset of the exponential phase, the pH_i_ which gradually decreased from pH = 7.4 to 7.2 at *t* = 11 h (**Figure 6A**). Twenty-five millimolars of K-acetate allowed a rapid increase in pH_i_ during germination. The pH_i_ during exponential growth remained stable at 7.2 during the experiment (**Figure 7A**).

High concentrations of WOAs were selected to reduce growth by 85%. P_ptsG_-driven expression of IpHluorin is delayed under these conditions, while spore-specific IpHluorin can be observed for longer periods of time because the signal is not diluted out. K-sorbate (10 mM) is shown to delay the maximum drop in OD_600_ indicative for spore germination. The data in **Figure 6B** show a drop from OD_600_ 0.13 to 0.08 in 216 min rather than from 0.13 to 0.07 in 84 min as was seen in the control shown in **Figure 5**. The rise of the pH_i_ was here similarly delayed as was the case with 3 mM K-sorbate. Such effects were not seen with 80 mM K-acetate, although the reduction in growth rate is similar (**Figure 7B**).

**FIGURE 8:**
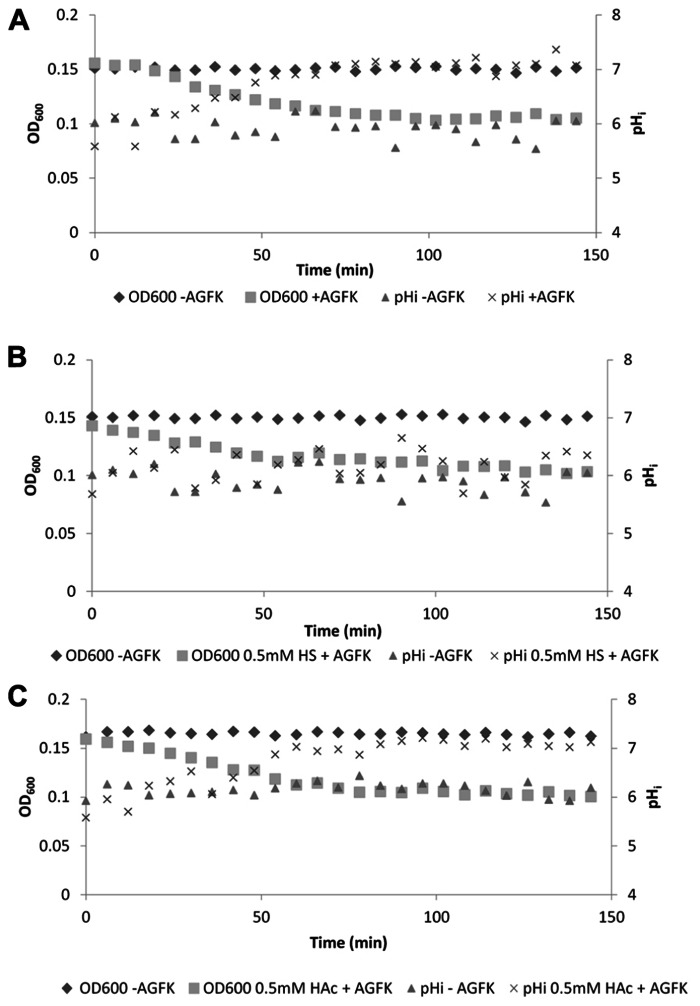
**OD_600_ and internal pH during germination of *B. subtilis* PB2 PsspE-IpHluorin spores in medium without glucose (pHo = 6.4).** Data are from a representative example. **(A)** Germination with AGFK; **(B)** germination with AGFK in 0.5 mM sorbic acid; **(C)** germination with AGFK in 0.5 mM acetic acid.

To further confirm the observation that sorbic acid inhibited the development of a positive inside pH gradient, spores of *B. subtilis* PB2 P_sspE_-IpHluorin were incubated with identical concentrations of either sorbic or acetic acid in medium without glucose other than present as germinant. When germination was triggered by addition of AGFK, spores incubated with sorbic acid showed a clear concentration dependant reduction in OD_600_ drop-rate as well as a reduced pH_i_ increase-rate. The OD drop-rate decreased from 80 × 10^-^^3^ to 40 × 10^-3^ OD_600_/min when 0.5 mM undissociated sorbic acid was present (**Figure 8B**). Such effects were not seen with acetic acid at identical concentrations, which behaved virtually identical to non-stressed germinating spores (**Figures 8A,C**). These observations are in agreement with earlier reports stating that sorbic acid can specifically inhibit germination of *B. cereus* and *B. subtilis*, likely by interacting with germinant receptors (Cortezzo et al., 2004; Van Melis et al., 2011).

## DISCUSSION

We show here that IpHluorin is an accurate, versatile probe to investigate the pH_i_ of *B. subtilis*. We were able to improve expression of pHluorin by fusion of the first 24 bp of *comGA* with the pHluorin-encoding gene. Genomic integration of IpHluorin resulted in more homogeneous expression levels compared to a multi-copy plasmid. It also resulted in a more stable construct, not requiring antibiotics for maintenance of the IpHluorin gene during extended periods of growth (not shown). The use of genomically integrated constructs with endogenous promoters for the expression of IpHluorin resulted in a strong enough signal for accurate pH measurements during exponential growth on glucose as well as compartment-specific pH_i_ measurements during sporulation. The IpHluorin that accumulates in the spore under control of P_sspE_ allows pH_i_ measurements of the *B. subtilis* spore. During spore germination and outgrowth, the signal from IpHluorin, expressed from P_sspE_ overlaps slightly in time with P_ptsG_-IpHluorin expression, thus allowing continuous pH_i_ monitoring during germination and outgrowth in batch. The pH values we have observed here closely resemble those found with other methods. During exponential growth, the pH_i_ approaches pH = 8. The pH_i_ of *B. subtilis* spores was also found to lie at approximately pH = 6. Despite the fact that expression levels of IpHluorin are much lower in spores, the pH value observed again closely corresponds to earlier reported values. The notion that during outgrowth a pH is observed that closely resembles the pH_i_ during exponential growth (as observed with P_ptsG_-IpHluorin) further corroborates the accuracy of our method.

Other methods to measure pH_i_ generally involve compounds that are hydrophobic and have WOA groups and may act as uncouplers, thereby depleting the ΔpH and influencing ΔΨ over the membrane. They are also more labor-intensive when high temporal resolution is required and except for fluorescent dyes do not allow cell type-specific pH measurements. However, these methods require long-term incubation with the dye plus extensive washing, taking up to 20 min to prepare the sample. Future studies will have to determine the phototoxicity and bleach rate of IpHluorin in individual (growing, sporulating, and germinating) cells.

We have observed clear differences in pH_i_ between P_ptsG_-IpHluorin and sporulation-specific IpHluorin. It has been shown that within a growing population of *B. subtilis* cells, differentiation occurs (Veening et al., 2006a, b) and this may affect metabolic state and pH_i_. This heterogeneity cannot be clearly monitored in batch without the use of more specific promoters or single cell observations. Also during spore germination such heterogeneity is seen (Smelt et al., 2008), so our results show the average of a germinating population.

During spore germination, the pH_i_ increases due to release of protons (Swerdlow et al., 1981). This process follows the drop in OD_600_, and results from H_2_O uptake and release of DPA. Our results show that a ΔpH is established rapidly. Such an increased pH can reactivate PGM, thus allowing the utilization of the spore’s 3-PGA store (Magill et al., 1994).

Taken together, our results show accurate, long-term pH_i_ monitoring in growing and sporulating *B. subtilis* cultures as well as during spore germination. The pH_i_ of sporulating cells is as high as that of exponentially growing cells. This is particularly the case for the mother cell. The pre-spore pH_i_ drops to pH = 6.0, however. The P_ptsG_-IpHluorin strain can be used for many experiments where the pH_i_ needs to be measured in cells growing on glucose, without the need for additional inducers like IPTG. Also, antibiotics are not strictly necessary. The sporulation-specific IpHluorin-expressing strains may give more insight in compartmentalization during sporulation, while the P_sspE_-IpHluorin strain may also help understanding spore germination characteristics in the presence of potential outgrowth inhibitors such as the WOAs sorbic acid and acetic acid.

Clearly, because not all cells are in exactly the same state, these data represent the average value of the pH_i_ in the population studies. To analyze the heterogeneity single-spore pH_i_ measurements are needed. Currently we are extending our single cell live imaging tool “SporeTracker” (Pandey et al., 2013) to that end.

## Conflict of Interest Statement

The authors declare that the research was conducted in the absence of any commercial or financial relationships that could be construed as a potential conflict of interest.
